# Effects of computerized decision support on maternal and neonatal health-worker performance in the context of combined implementation with performance-based incentivisation in Upper East Region, Ghana: a qualitative study of professional perspectives

**DOI:** 10.1186/s12913-022-08940-0

**Published:** 2022-12-26

**Authors:** Gifty Apiung Aninanya, John E Williams, Afua Williams, Easmon Otupiri, Natasha Howard

**Affiliations:** 1 Department of Health Services Policy, Planning, Management and Economics, School of Public Health, University for Development Studies, Box TL 1350, Tamale, Ghana; 2 Dodowa Health Research Centre, PO Box DD1, Dodowa, Ghana; 3 Ga North Municipal Hospital, Ghana Health Service, Accra, Ghana; 4 Kwame Nkrumah University of Science and Technology, Kumasi, Ghana; 5 Saw Swee Hock School of Public Health, National University of Singapore and National University Health System, 12 Science Drive 2, Singapore , 117549 Singapore; 6 Department of Global Health and Development, London School of Hygiene and Tropical Medicine, 15-17 Tavistock Place, London, WC1H 9SH UK

**Keywords:** Computerised-decision support system, Performance-based incentives, mHealth, Maternal and neonatal health, Ghana

## Abstract

**Background:**

Computerized decision support systems (CDSS) and performance-based incentives (PBIs) can improve health-worker performance. However, there is minimal evidence on the combined effects of these interventions or perceived effects among maternal and child healthcare providers in low-resource settings. We thus aimed to explore the perceptions of maternal and child healthcare providers of CDSS support in the context of a combined CDSS-PBI intervention on performance in twelve primary care facilities in Ghana’s Upper East Region.

**Methods:**

We conducted a qualitative study drawing on semi-structured key informant interviews with 24 nurses and midwives, 12 health facility managers, and 6 district-level staff familiar with the intervention. We analysed data thematically using deductive and inductive coding in NVivo 10 software.

**Results:**

Interviewees suggested the combined CDSS-PBI intervention improved their performance, through enhancing knowledge of maternal health issues, facilitating diagnoses and prescribing, prompting actions for complications, and improving management. Some interviewees reported improved morbidity and mortality. However, challenges described in patient care included CDSS software inflexibility (e.g. requiring administration of only one intermittent preventive malaria treatment to pregnant women), faulty electronic partograph leading to unnecessary referrals, increased workload for nurses and midwives who still had to complete facility forms, and power fluctuations affecting software.

**Conclusion:**

Combining CDSS and PBI interventions has potential to improve maternal and child healthcare provision in low-income settings. However, user perspectives and context must be considered, along with allowance for revisions, when designing and implementing CDSS and PBIs interventions.

## Background

Frontline health-worker performance is a common challenge in resource-constrained settings [1]. Health-worker behaviours are complex with many potential influences, though research indicates general enablers of improved performance include in-service training in use of tools, constructive feedback, and receiving positive recognition [2]. In low and middle-income country (LMIC) health systems, performance is additionally constrained by staffing deficiencies and ad hoc task shifting thus weakening capacity to achieve national and global health goals [3]. Access to health infrastructure is indispensable but not enough [4] and poor quality-of-care impedes achieving Sustainable Development Goal 3 ‘ensure healthy lives and promote well-being for all at all ages’ [5]. Therefore, health systems must ensure good quality care.

A key example of this issue is maternal and neonatal health (MNH), which cannot improve in LMICs without ensuring the availability of adequate numbers of competent and motivated providers at health facilities. Despite improvements in access to and use of MNH care in many LMICs, mortality rates remain high and gaps between low-income and high-income countries continue [6]. The high levels of mortality are attributed to poor quality of care in the health facilities [6–9], characterized by overcrowding and understaffing, with inappropriate management of complicated deliveries and untimely response to emergencies [10].

Initial efforts focused on improving MNH provider performance through in-service trainings, assuming problems are due to inadequate knowledge and skills [11]. Millions of training dollars later, healthcare indicators are little improved and MNH providers still require support [11]. Recognising training is not the only solution, more recent interventions involve infrastructure improvements and advocacy for skilled birth attendance [12, 13]. Packages of interventions appear more effective than single interventions [1, 8]. For example, assessment of Standards-Based Management and Recognition (SBM-R©) for quality improvement, in three Ethiopian hospitals and eight health centres, showed significant improvement in MNH provider performance during labour, delivery, and immediate postnatal services [14].

Evidence suggests computerised decision-support systems (CDSS) and performance-based incentives (PBIs) could enhance health-worker motivation and performance [15–17]. CDSS are electronic tools that prompt provider behaviours with patient-specific prompts, treatment guidelines, automatic medication dosing calculations, and reports of overdue tests and medications [18]. PBIs involve compensating health-workers for work beyond their typical remit so they feel appreciated and that effort is valued. Offering monetary or other recognition for high-performing employees aims to counteract weak or indirect health system incentives [19, 20] by directly linking rewards to actions that achieve performance targets and therefore contribute to improved health outcomes [16, 21, 22]. PBIs can also enhance effectiveness by stimulating innovation, enhancing teamwork, incentivizing service in remote locations, and improving retention [15, 23–25].

Literature focuses on individual effect of PBIs on health-worker motivation but not on the combined effects of CDSS and PBIs on performance [15, 26]. Our previous study in Ghana’s Upper East Region was one of the first to analyse the combined effects of these interventions on client satisfaction with maternal and neonatal healthcare [16]. Implementation of CDSS in the context of an ongoing PBI intervention in Upper East Region provided an opportunity to examine perceived effects of both interventions among health-workers, as we found no other qualitative studies on this topic and research had shown sub-optimal motivation and performance among maternal and neonatal healthcare providers in these areas and potential scope for improvement [25, 27]. This qualitative study thus aimed to explore health-worker perspectives of the effects of CDSS, in the context of a combined CDSS-PBI intervention, on maternal and child healthcare provision in primary facilities in Ghana’s Upper East Region.

## Methods

### Study design and sites

This qualitative study reanalyses 66 semi-structured interviews conducted with frontline health-workers, facility managers, and district supervisors at the end of the European Union-funded Quality of maternal and neonatal health (QUALMAT) intervention trial [15, 26, 28]. This cluster-randomized un-blinded controlled trial, conducted in Ghana’s Upper East region, aimed to assess the effect of a CDSS for maternal and neonatal health care services.

The trial was conducted in twelve health facilities in Kassena-Nankana (KND) and Builsa districts and is described in Aninanya et al. [16]. KND (intervention district) has an estimated population of 152,000 served by one hospital in the district capital Navrongo, six health centres, one private clinic, and twenty-seven Community-based Health Planning and Services (CHPS) compounds. Builsa (comparison district) has an estimated population of 95,800 served by the district hospital in Sandema, six health centres, and thirteen CHPS compounds. MNH services were considered poor in Upper East Region, due to insufficient and demotivated staff; inadequate access to and use of computers, reproductive health guidance, and protocols; and inadequate diagnoses and referrals [29–31]. A cross-sectional study of 400 new mothers indicated 93% had delivered in health facilities and 97% had received antenatal care with 75% having four or more antenatal visits [32]. However, institutional MMR in the Upper East Region prior to the trial was 352 per 100,000 live births and estimated at 367 and 259 per 100,000 live births In KND and Builsa district respectively [33]. Nurses and midwives are responsible for provision of facility-based MNH services in these districts [34], with low productivity reported as some providers rarely used treatment guidelines or partograph, and missed health education opportunities [8].

CDSS and PBI interventions were implemented in six KND intervention facilities for two years (2012–2014). CDSS included computerized clinical support in implementing WHO ‘Pregnancy, Childbirth, Postpartum and Newborn Care: a guide for essential practice’ guidelines for 35 purposively selected and trained midwives, community health nurses, and facility managers. Each intervention facility received a laptop, with CDSS software and IT support, shared between three trained midwives and nurses, while facility managers were tasked with supervision.

Performance was rewarded based on improvements within facilities rather than in comparison to other facilities. Individuals were identified for PBIs based on achieving key indicators determined by facility leadership, regional and district health directorates, and the PBI committee. PBI consisted of rewarding best-performing midwives and facilities with domestic appliances (e.g. blenders, fans, stoves, freezers, fridges, televisions, saucepans, cloths, kettles, microwaves) and recognition certificates at annual awards ceremonies, along with regular supervision, verbal appreciation, furniture, and small monthly allowances [15, 16]. Key performance indicators included proportion of ANC visits recorded in CDSS; proportions of pregnant women receiving tetanus vaccination, iron supplementation, safe sex and HIV counselling; proportion of births attended by skilled personnel; proportion of partographs completed in CDSS; partograph usage rate; proportion of women referred based on CDSS recommendation; and proportion of newborns vaccinated [16].

### Sampling and recruitment

We included all 24 participating CDSS users, 6 facility managers, and 6 district-level staff in intervention facilities and used purposive non-probability sampling to select equal numbers of equivalent participants in comparison facilities. This enabled us to include non-intervention participants with similar workloads who could provide relevant insights. GAA obtained written informed consent from all participants before interview.

### Data collection

We developed an interview guide from the literature and expert opinion. Topics included basic demographics, experiences of CDSS and PBI interventions if relevant, work challenges, and suggested solutions. GAA and two research assistants conducted in-person audio-recorded interviews in English, lasting 35–45 min, in locations such as homes and health facilities as chosen by participants. We used interviews with facility managers and district-level staff, because of their varied involvement supervising the intervention, to triangulate and crystalize findings from frontline providers. To ensure confidentiality, we assigned identification codes and did not include personal identifiers in study tools or outputs. Audio files were transcribed verbatim by two professional transcriptionists.

### Analysis

GAA imported transcripts into Nvivo 10 (QSR International Pty Ltd, Victoria, Australia) data management software [35] and analysed them thematically using Braun and Clarke’s six-stage approach [36]. In summary, GAA read and became familiarised with the data, identified deductive codes based on interview guide topics and inductively coded new or unexpected topics arising in transcripts. She developed a coding structure iteratively, collating codes related to intervention effects, challenges, and suggestions into themes. GAA examined relationships between codes, compiled and summarised contents of each theme with support from co-authors, and conducted thematic mapping. GAA and NH refined and defined themes and sub-themes through discussion, further integration, and the reporting process. Triangulation of participant perspectives (i.e. midwives, nurses, medical assistants, district public health nurses, directors) at different health system levels and in both study arms helped improve validity.

## Results

### Participant characteristics and analytical themes

In all 66 participants were included in the study, 32 in intervention and 34 in comparison facilities. Most (64) were women, 48 were maternal healthcare providers (i.e. 24 each intervention and comparison), 12 were facility managers (i.e. 6 each intervention and comparison), and 6 were district-level staff (i.e. 2 intervention and 4 comparison).

We organised findings under three deductive themes of perceived CDSS-PBI effects on provider performance, challenges to CDSS use, and suggestions for improving provider performance. We reported perceptions of frontline and managerial participants in intervention and control facilities separately and included inductive sub-themes as appropriate.

### CDSS-PBI effects on provider performance

All 36 intervention participants indicated CDSS-PBI interventions had enhanced performance. Reportedly, MNH care providers’ knowledge of maternal health issues increased and quality-of-care improved. CDSS helped guide diagnoses and prescribing, prompting health-workers on necessary actions in managing routine care and pregnancy-related complications. Three inductive sub-themes were: (i) reminders and health education support, (ii) improved performance, and (iii) improved clinical processes and outcomes.

#### Reminders and health education support

Midwives and nurses generally appreciated the automatic reminders, such as to provide deworming drugs.

*“It alerts us on what to do and because of that, we can do things. For the Albendazole [*deworming*], that one if you have not given to the woman and you are entering it will ask you whether you have given the drug […]. It has affected IPT [*intermittent preventive treatment for malaria*] because the way the book is, sometimes you can forget that the person has not taken IPT, but the computer will alert you…”* (Midwife, 34 years, intervention arm)

*“I will say the quality of maternal care has improved. We are now prompted to administer certain drugs like Albendazole…”* (Midwife, 60 years, intervention arm)

Similarly, many frontline providers noted how useful it was having key messages and educational information at their fingertips.

“*It has helped me to know more. Especially, if you are educating an ANC woman, you can just educate the woman without cracking your brains. The information we get from the CDSS to educate the women has helped improve the quality of care.”* (Midwife, 33 years, intervention arm)

Generally, while several noted that it could not replace skilled midwifery decision-making, most noted that they were pleased to get reminders.

*“[I]t will be alerting you on what to do. So, I will say it guides us on what to do and how to do but before, you have to make your own decision.”* (Midwife, 33 years, intervention arm)

#### Improved performance

Several frontline health-workers described being more efficient or better performing their duties. Some described this in relation to CDSS.

*“It [CDSS] has affected antenatal coverage because you are able to attend to more clients in a day. Sometimes we attend to about 50 women in a day…”* (Midwife, 34 years, intervention arm)

*“Performance has improved in the sense that if you open the 2012 and the 2013 registers, you will see a vast difference. Now we check all the HB* [haemoglobin] *of the clients and we also counsel them a lot. Also, if the woman comes and she is 28 weeks, the computer will tell you what to do since it is pre-term labour.”* (Medical Assistant, 54 years, intervention arm)

Many described improvements in relation to PBIs and increased motivation.

“*I work hard since I know it will pay off by not only improving quality of care but something small from the project*…” (Midwife, intervention facility)

Some midwives also related PBIs to improving their job satisfaction as well as performance.

*“I like the performance-based package because when I perform well, I am awarded. I get inner satisfaction. That helps me to give my best.” (Midwife, intervention facility)*

Managers with access to aggregated data described similar perspectives.

*“The interventions [CDSS-PBI] have contributed to improved performance because the midwife is more committed to work. She barely even leaves the maternity and I think it has even increased some of the indicators and deliveries over the past few years. Our facility has contributed significantly to the district performance, which has been recognized by the district. There has been an improvement in anti-tetanus vaccinations to pregnant women and intermittent preventive treatment. I realized that previously there were issues of IPT3, child welfare and Penta 3 but I think over the past 2 years there has been an improvement.”* (Medical Assistant, 37 years, male, intervention arm)

Several managers also noted benefits of PBIs, particularly in terms of midwives’ motivation to work more actively.

*“Performance-based incentives have made them happy. It shows that they are working. You know they had certificates, they had parcels […]. This has made them very happy, and they are doing the work. It has made them to do the work more and more.”* (Facility manager, intervention facility)

#### Improved clinical processes and outcomes

Most described improved work processes, with some describing this as improving quality-of-care while others referred to improving MNH outcomes, including mortality. For example, a district manager referred to CDSS as their new doctor:

*“The computer also has all the protocols so, because this district we don’t have a doctor, the computer is now our doctor. The computer guides us. The nurse doesn’t panic because the computer tells them what to do.”* (District Public Health Nurse, 54 years, intervention arm)

Frontline providers similarly described how protocols and reminders improved clinical processes for them.

*“With the CDSS, if a client’s BP [blood pressure] is high, it will ask you to recheck and if it is still high, it will ask you to do something for the client, either you refer or detain. With the paper-based protocols, most often you just skip these vital things. CDSS also makes it easy to access previous patient’s history.”* (Midwife, 27 years, Intervention arm).

*“For PNC [postnatal care], after delivery you have to check the woman frequently; 15 min, 30 min, 1 h for 6 h and at the end you can know the woman is now well.”* (Midwife, 34 years, intervention arm)

Many health-workers connected process improvements with improved maternal outcomes, either through women receiving all required services and treatments or through encouraging earlier referrals.

*“It has reduced maternal deaths because if a woman comes and you monitor to a certain level, the CDSS will just tell you to refer and so it makes us to refer early.”* (Midwife, 44 years, intervention arm)

Municipal and district managers had similar perspectives on the advantages of CDSS. As one noted:

*“…with the paper-based protocols, they are mostly in their cabinets and not on their tables and that make them feel lazy using them, but this one makes you alert as you are entering the data while reviewing the protocol. Without the protocol, some even refer cases that they are not supposed to refer… The CDSS has improved the midwives’ knowledge and helps them refer clients early. Quality of maternal healthcare has improved because you don’t do things in the abstract.”* (Municipal Public Health Nurse, 58 years, intervention arm)

Several managers also described specific benefits of PBIs. As another noted:

*“Health workers were behaving negatively towards work but the [PBI] intervention has come to improve on it. For the first time it was introduced they were not serious because they didn’t know there will be awards but when the first award ceremony came, they became serious and when they go to work, they are actually busy…’* (District Public Health Nurse, 59 years, intervention arm)

### Challenges to CDSS use

Alongside perceived improvements, intervention participants described challenges in using CDSS. Some appeared relatively easily addressable, such as software requiring administration of only one malaria IPT to pregnant women and increased workloads as nurses and midwives still had to complete facility forms in addition to CDSS data screens. Others were more challenging, such as faulty electronic partograph leading to unnecessary referrals and power fluctuations affecting software use. The IPT gap was a common complaint.

*“There are certain things in the CDSS that should be improved. The IPT should be first, second, and third. It’s only once in the CDSS*” (Midwife, 54 years, intervention arm).

Most frontline health-workers found it annoying and time-consuming to complete both CDSS data screens and paper documentation.

*“It has also increased our workload because we have to use the computer, and attend to the woman, and at the same time enter it into the book”* (Midwife, 29 years, intervention facility).

Some midwives suggested that maternity clients also found this annoying.

*“The only complaint they (clients) give is that they keep long because you enter their personal information into the maternal book and afterwards you enter the same information into the CDSS”* (Midwife, 33 years, intervention arm).

Some also suggested that added form filling distracted from patient care.

*“In fact, to use the computer to monitor a patient is sometimes difficult, it doesn’t need one person. If a woman is in labour, you can’t come to the computer again”* (Midwife, 56 years, intervention arm).

Many health-workers noted that the CDSS partograph required too many referrals.

*“If you want to follow the partographs strictly on the CDSS, you will end up referring all your delivery clients” (Midwives, 29 years, intervention arm).*

All noted that CDSS equipment’s short battery life and reliance on electricity could be problematic.

*“Sometimes too, if there is light out and the battery is down you can’t use it for patient care”* (Midwife, 40 years, intervention arm).

### Suggestions for improving provider performance

Other than addressing the issues highlighted above, suggestions for improving performance came from comparison arm participants. Interestingly, all comparison participants reported some performance improvements (the study was not blinded) but noted considerable need for further improvement. However, when asked whether they knew anything about CDSS or PBIs or how these worked most said no and were thus not questioned further about the intervention. Instead, the issues they discussed were primarily about the challenges they faced in their work and how these could be addressed. Despite their lack of reported knowledge, all comparison health-workers requested PBIs, along with more promotion opportunities and staff to spread the workload, and described the need for protocols, phone-based guidelines, and equipment (e.g. test kits, blood pressure apparatus, haemoglobin machines) to enhance their work.

*“There is a slight improvement in performance but there is still more room for improvement. Protocols and incentives that are supposed to enhance our performance are inadequate.”* (Midwife, 48 years, comparison arm)

Comparison area managers reiterated frontline perspectives, with poor partograph use in comparison facilities highlighted.

*“Performance is moderate because we don’t have the requisite protocols in the facility to make clinical decisions. Partograph utilization is poor in most cases.”* (Community health officer, 27 years, comparison arm)

*“Generally, the performance of nurses is encouraging. Although some indicators have declined, it is still not bad. Partograph utilization is poor but labour and delivery services, tetanus care coverage, and pregnancy at risk referred seem to be looking good based on the statistics.”* (District Health Information Officer, 41 years, comparison arm)

All comparison health-workers advocated for access to CDSS.

*“Authorities should introduce electronic decision support systems to enhance patient-based care”* (Midwife, 57 years, comparison arm).

Comparison managers expressed the same wish to have the CDSS-PBI intervention in their facilities.

*“All facilities should have computers where the providers can also learn new protocols in maternal and neonatal health services through the internet. They should be trained on how to use these computers to monitor labour and they should be given financial package.”* (District health information officer, 37 years, comparison arm)

*“I will suggest improved logistics and human resources, provision of financial package to both clients and workers to boost their morale.”* (District Director, 59 years, comparison arm)

## Discussion

This qualitative study examined frontline and managerial perspectives of CDSS combined with a PBI intervention, indicating perceived improvements in health-worker performance and conditions as compared to comparison arm counterparts. CDSS appeared to enhance provider knowledge of maternal health issues and health education messaging, support care provision through reminders and protocols, and increase referrals. This was despite baseline research suggesting very limited technological knowledge among these providers [28, 37]. Several intensive trainings and supportive supervision equipped health-workers with the necessary technical skills to use computers for maternal care, as also shown in Canada [38]. This reinforces research findings on the benefits of CDSS for MNH provider performance [28, 39] and implies that contributions to achieving SDG 3 and universal health coverage (UHC) [40] could invest in such interventions to enhance health-worker performance [12, 34, 41, 42].

Comparison area participants, on the contrary, reported commensurately smaller improvements with suggestions for additional changes that likely related to other simultaneous MNH initiatives in Upper East region [16, 43, 44]. Interestingly, suggestions for improvement were all from comparison facilities and despite indicating their lack of awareness of CDSS and PBIs they expressed considerable interest. This suggests that the concepts of CDSS and PBIs were quite popular with or without explicit knowledge of either intervention. It is thus worth noting that comparison participants recommended CDSS-PBI intervention components [40] be introduced in their facilities to further improve performance (e.g. protocols on their mobile phones, incentives), along with essential supplies and equipment such as test kits, blood pressure apparatus. This supports evidence from other settings on the perceived benefits of CDSS and PBIs to improve performance [2, 25, 37].

While it was not possible to confirm the maternal mortality improvements reported by participants, recent evidence showed this intervention improved antenatal and delivery clients’ satisfaction with care [16]. Increased satisfaction suggests services, attitudes, or both had improved [45]. Additionally, use of relevant clinical guidelines was reportedly high among intervention health-workers, while earlier research in the area showed this as poor [27, 31]. Thus, improved access to and required use of clinical protocols and guidelines as part of CDSS in intervention facilities likely meant improved quality-of-care and reduced clinical errors, as indicated in other research on use of computerized guidelines to reduce errors [46] and epidemiological research in Ghana and other LMICs [46, 47].

What this study does show is that, despite some operational challenges (e.g. software glitches, electricity), health-workers’ reported job satisfaction and quality-of-care improved with the introduction of the CDSS-PBI intervention. This is promising, though further research is needed on intervention cost-effectiveness and sustainability before any policy decision should be taken on wider implementation.

### Strengths and limitations

The study fills a gap in knowledge as there are no studies on the combined effects of these interventions on health-workers care providers’ performance. However, several limitations should be considered when interpreting the findings. First, while the focus was on CDSS, both CDSS and PBI were running concurrently, it was not feasible to assess the two intervention components separately. Second, the qualitative results present just the perceptions of health professionals with regards to the intervention, a mixed-method approach would have created an opportunity to collect both qualitative and quantitative data at the same time and would have offset weaknesses associated with using one method [48].

## Conclusion

Findings indicated predominantly positive perceptions of the CDSS-PBI intervention helped improve MNH care provider performance in the Upper East Region. CDSS and PBIs have potential to improve frontline health-workers’ motivation and adherence to clinical guidelines. However, while this combined intervention shows promise for longer-term or wider implementation, context and perspectives must be considered when developing, implementing, and assessing any performance improvement intervention. Thus, further research is still required to assess the cost-effectiveness and sustainability of performance improvements.

## Data Availability

Study datasets can be shared by the corresponding author on reasonable request.
